# The effect of active and passive smoking during pregnancy on birth outcomes: A cohort study in Shanghai

**DOI:** 10.18332/tid/188866

**Published:** 2024-07-01

**Authors:** Xiaokai Wang, Xia Gao, De Chen, Xuelian Chen, Qingwei Li, Jiani Ding, Fangyuan Yu, Xiaoyun Zhu, Nannan Zhang, Yifang Chen

**Affiliations:** 1 Department of Shanghai Jinshan District Disease Prevention Center, Shanghai, People's Republic of China; 2 Shanghai Health Promotion Center, Shanghai, People's Republic of China; 3 Department of Shanghai Jinshan District Maternity and Child Care Centers, Shanghai, People's Republic of China

**Keywords:** pregnancy, smoking, birth outcomes, cohort study

## Abstract

**INTRODUCTION:**

China is the largest tobacco consumer in the world, and tobacco poses a serious threat to the health of pregnant women. However, there are relatively few domestic studies on smoking during pregnancy and childbirth outcomes among pregnant women. The purpose of this study was to analyze the effect of active and passive smoking on pregnant women and their pregnancy outcomes, providing evidence and recommendations for intervention measures.

**METHODS:**

This was a cohort study in Shanghai from April 2021 to September 2023. According to the smoking status of pregnant women, they were divided into three groups: active smokers, passive smokers and non-smokers. A self-designed questionnaire was utilized to conduct the survey, and their pregnancy outcomes were tracked and followed up.

**RESULTS:**

A total of 3446 pregnant women were included in this study, among which 2.1% were active smokers, 43.5% were passive smokers, and 54.4% were non-smokers. The average age of the pregnant women was 29.9 years, and 41.2% had a university degree or higher. The education level of active smokers and passive smokers was significantly lower than that of non-smokers (p<0.05).The average gestational age of non-smokers was 38.6 weeks, and the birth weight was 3283.2 g, which was higher than those of active smokers and passive smokers (p<0.05). Logistic regression analysis showed that passive smoking increased the likelihood of preterm birth (AOR=1.38; 95% CI: 1.05–1.81), low birth weight (AOR=1.53; 95% CI: 1.10–2.12), and intrauterine growth restriction (AOR=1.35; 95% CI: 1.02–1.79), while active smoking increased the likelihood of preterm birth (AOR=2.98; 95% CI: 1.50–5.90), low birth weight (AOR=4.29; 95% CI: 2.07–8.88), intrauterine growth restriction (AOR=2.70; 95% CI: 1.37–5.33) , and birth defects (AOR=2.66; 95% CI: 1.00–6.97).

**CONCLUSIONS:**

Our findings illustrate that active and passive smoking can lead to adverse pregnancy outcomes. This study provides data on the relationship between smoking during pregnancy and delivery outcomes among pregnant women. In the future, we need more effective strategies to protect pregnant women from the harm of tobacco.

## INTRODUCTION

China is the world’s largest producer and consumer of tobacco, accounting for more than one-third of tobacco consumption worldwide^1^. China’s high tobacco consumption rate has led to significant health burdens. Among Chinese adults, smoking is associated with increased risks of morbidity and mortality from a wide range of diseases^2^. Although the majority of smokers in China are male, the prevalence of smoking among pregnant women still reaches 3.8%. Given China’s large population, this percentage still accounts for a very large number of people. In addition, the prevalence of smoking among reproductive-age women aged <40 years has increased significantly in recent years^3-5^. Another national prevalence survey revealed that more than 50% of Chinese women of childbearing age are exposed to secondhand smoke, a rate far above the world average^6^.

Active smoking during pregnancy involves the direct inhalation of tobacco smoke, which exposes both the mother and fetus to a high concentration of harmful substances. The toxic compounds in cigarette smoke have been shown to cause vasoconstriction, inflammation, and oxidative stress, all of which can contribute to complications such as preterm birth, low birth weight, and intrauterine growth restriction. Additionally, these substances can cross the placenta and directly affect the developing fetus, increasing the risk of birth defects^7,8^.

Passive smoking during pregnancy exposes the fetus to harmful substances present in secondhand smoke, including nicotine, carbon monoxide, and other toxic chemicals. These substances can disrupt fetal development, impair placental function, and restrict the supply of oxygen and nutrients to the fetus, leading to adverse outcomes^9,10^.

The prenatal period is a crucial time for maternal and infant health, and maternal active and passive smoking negatively affect the quality of life of mothers and infants in China^11^. Given the impact of tobacco on the health of pregnant women, there is an urgent need to gain a thorough and quantitative understanding of active and passive smoking among pregnant women, but there are relatively few studies on maternal smoking during pregnancy and birth outcomes in China. This study aimed to investigate the current status of tobacco exposure among pregnant women in Shanghai and its impact on pregnancy outcomes, providing evidence and recommendations for intervention measures.

## METHODS

### Participants

This is a cohort study conducted in Shanghai from April 2021 to September 2023. The participants were pregnant women who met the ‘Shanghai Maternal Health Manual’ criteria and gave birth in Shanghai. The inclusion criteria for this study were: pregnant women who had lived in Shanghai for more than 6 months and were willing to participate in the study. The exclusion criterion for this study was pregnancy complications.

This study was conducted in accordance with the World Medical Association Declaration of Helsinki. This study was approved by the Ethics Committee of the Shanghai Jinshan District Disease Prevention Center. All participants signed informed consent forms when completing the questionnaires. All the data used for analysis were anonymous.

### Data collection

A self-designed questionnaire was used after consulting the literature. The information collected by the questionnaire mainly included the following three aspects: 1) basic information about the pregnant women (age, education level, occupational status, reproductive history, etc.); 2) active and passive smoking among pregnant women (time, place, etc.); and 3) the cognition, attitudes and behaviors of the pregnant women towards smoking and passive smoking.

Pregnant women were regularly followed up, and their pregnancy outcomes, including newborn sex, birth time, gestational week, birth length, birth weight, delivery mode, birth defect status, and Apgar score, were assessed after delivery using medical records.

### Standard definitions

Pregnant women were considered active smokers if they have smoked in the past 30 days. Non-smoking pregnant women were considered passive smokers if they were exposed to tobacco smoke for >15 minutes at least one day per week. Non-smokers were defined as pregnant women with neither active nor passive smoking. Preterm infants were those born between 28 weeks and 37 weeks of gestation. Low-birth-weight infants were with a birth weight <2500 g at birth. Macrosomia was defined as a birth weight >4000 g. Growth restriction was defined as a birth weight below the 10th percentile of the mean weight for the same gestational age. Birth defects referred to various abnormalities at birth. Birth defect was defined as a physical or biochemical abnormality that is present at birth, such as heart defects, cleft lip and palate, etc.

### Quality control

The investigators in this study were uniformly trained and were able to consistently apply standardized methods. The questionnaires were collected and sorted by a specialized person. If the data missing in a questionnaire were ≥10%, the questionnaire was rejected. After delivery, medical records were checked by a specialized investigator. These investigators tracked pregnancy outcomes in detail based on medical records and conducted on-site quality control.

### Statistical analysis

Epidata 3.1 software was used for double data entry. The SPSS 26.0 statistical software package was utilized for the statistical analysis of the data. For the basic characteristics of the study participants, we employed descriptive analysis methods, which included calculating statistics such as means, standard deviations, and percentages. Depending on the characteristics of the data, different statistical analysis algorithms were adopted. When the data exhibited a normal distribution, we used the t-test. For non-normally distributed data, we applied the Mann-Whitney U test or the Kruskal-Wallis H test. When the data met the conditions of homogeneity of variance and normal distribution, we employed ANOVA to compare differences between multiple groups. For categorical data, we utilized the chi-squared test to compare differences in frequency distributions between different groups. In analyzing the pregnancy outcomes of the pregnant women, we applied a logistic regression model. To control for potential confounding variables, we adjusted the logistic regression model (adjusting for the effects of age, education level, occupational status, first pregnancy status, and first birth status.). The significance level for all statistical analyses was set at α=0.05.

## RESULTS

A total of 3446 pregnant women, including 73 active smokers, 1499 passive smokers, and 1874 non-smokers, were included in this study from April 2021 to September 2023. The average maternal age was 30.1 ± 4.9, 29.7 ± 4.3 and 30.1 ± 4.3 years among active smokers, passive smokers and non-smokers, respectively. Active smokers and passive smokers were significantly less educated than non-smokers (p<0.05). In addition, compared with passive smokers and non-smokers, active smokers had lower rates of first pregnancy and first birth status (p<0.05). The characteristics of the participants in the studied groups are presented in Table 1.

**Table 1:** Demographic characteristics of the pregnant women by smoking status, Shanghai, 2021–2023 (N=3446)

*Characteristics*	*Active smokers n (%)*	*Passive smokers n (%)*	*Non-smokers n (%)*	*Total n (%)*	*p*
**Total**	73 (2.1)	1499 (43.5)	1874 (54.4)	3446 (100)	
**Age (years)**, mean ± SD	30.1 ± 4.9	29.7 ± 4.3	30.1 ± 4.3	29.9 ± 4.4	0.247
**Education level**					<0.001
Lower than university	62 (84.9)	964 (64.3)	1033 (55.1)	2059 (59.8)	
University and postgraduate	11 (15.1)	535 (35.7)	841 (44.9)	1421 (41.2)	
**Occupational status**					0.410
Employed	37 (50.7)	828 (55.2)	1066 (56.9)	1931 (56.0)	
Unemployed	36 (49.3)	671 (44.8)	808 (43.1)	1515 (44.0)	
**Pregnancy age**					0.417
First trimester	66 (90.4)	1383 (92.3)	1745 (93.1)	3194 (92.7)	
Second trimester	7 (9.6)	111 (7.4)	118 (6.3)	236 (6.8)	
Third trimester	0 (0)	5 (0.3)	11 (0.6)	16 (0.5)	
**First pregnancy**					0.032
Yes	23 (31.5)	649 (43.3)	856 (45.7)	1528 (44.3)	
No	50 (68.5)	850 (56.7)	1018 (54.3)	1918 (55.7)	
**First birth**					0.038
Yes	27 (37.0)	721 (48.1)	948 (50.6)	1696 (49.2)	
No	46 (63.0)	778 (51.9)	926 (49.4)	1750 (50.8)	

Statistical tests: chi-squared test.

Table 2 shows the birth outcomes according to smoking status. There were no differences in infant sex, delivery mode, body length or Apgar score among women by smoking status (p>0.05). The average gestational weeks of active smokers, passive smokers, and non-smokers were 38.1, 38.5, and 38.6, respectively. Their average birth weights were 3141.0 g, 3263.3 g, and 3283.2 g, respectively. The gestational weeks and birth weights of non-smokers were higher than those of active smokers and passive smokers (p<0.05).

**Table 2:** Birth outcomes of the pregnant women by smoking status, Shanghai, 2021–2023 (N=3446)

*Characteristics*	*Active smokers n (%)*	*Passive smokers n (%)*	*Non-smokers n (%)*	*Total n (%)*	*p*
**Total**	73 (2.1)	1499 (43.5)	1874 (54.4)	3446 (100)	
**Infant sex**					0.319
Male	33 (45.2)	768 (51.2)	991 (52.9)	1792 (52.0)	
Female	40 (54.8)	731 (48.8)	883 (47.1)	1654 (48.0)	
**Mode of delivery**					0.584
Vaginal	32 (43.8)	708 (47.2)	919 (49.0)	1659 (48.1)	
Instrumental	0 (0)	17 (1.1)	17 (0.9)	34 (1.0)	
Cesarean	41 (56.2)	774 (51.6)	938 (50.1)	1753 (50.9)	
**Gestational age**, mean ± SD	38.1 ± 1.8	38.5 ± 1.6	38.6 ± 1.5	38.5 ± 1.5	0.019
**Body length** (cm), mean ± SD	49.7 ± 1.3	49.9 ± 1.4	49.9 ± 1.4	49.9 ± 1.4	0.421
**Birth weight** (g), mean ± SD	3141.0 ± 534.2	3263.3 ± 471.4	3283.2 ± 454.6	3271.54 ± 464.2	0.024
**Apgar score,** mean ± SD	9.5 ± 0.6	9.5 ± 0.8	9.6 ± 0.7	9.6 ± 0.8	0.256

Statistical tests: chi-squared test.

Logistic regression analysis was used to evaluate the effects of passive smoking on pregnancy outcomes after adjusting for the effects of age, education level, occupational status, first pregnancy status, and first birth status. Table 3 shows that the odds of premature birth were significantly increased in passive smokers compared to non-smokers (AOR=1.38; 95% CI: 1.05–1.81, p=0.021). The odds of low birth weight were significantly increased in passive smokers compared to non-smokers (AOR=1.53; 95% CI: 1.10–2.124, p=0.011). The odds of growth restriction were significantly increased in passive smokers compared to non-smokers (AOR=1.35; 95% CI: 1.02–1.79, p=0.039).

**Table 3:** Effects of passive smoking on adverse pregnancy outcomes, Shanghai, 2021–2023 (N=3446)

*Characteristics*	*Passive smokers (N=1499)*	*Non-smokers (N=1874)*	*OR (95% CI)*	*AOR (95% CI)*	*p*
Premature birth	116	107	1.39 (1.06–1.82)	1.380 (1.050–1.814)	0.021
Low birth weight	86	70	1.57 (1.14–2.17)	1.530 (1.10–2.12)	0.011
Macrosomia	70	99	0.88 (0.64–1.20)	0.879 (0.64–1.21)	0.424
Growth restriction	106	99	1.36 (1.03–1.81)	1.350 (1.02–1.79)	0.039
Birth defect	24	47	0.63 (0.39–1.04)	0.63 (0.38–1.03)	0.066

AOR: adjusted odds ratio; adjusted for maternal age, education level, occupational status, first pregnancy status, and first birth status.

The effects of active smoking on adverse pregnancy outcomes are shown in Table 4. Compared with non-smokers, smokers had significantly greater odds of premature birth (AOR=2.98; 95% CI: 1.50–5.90, p=0.002), low birth weight (AOR=4.29; 95% CI: 2.07–8.88, p<0.001), growth restriction (AOR=2.70; 95% CI: 1.37–5.33, p=0.004), and birth defects (AOR=2.66; 95% CI: 1.00–6.97, p=0.049).

**Table 4:** Effects of active smoking on adverse pregnancy outcomes, 2021–2023 (N=3446)

*Characteristics*	*Active smokers (N=73)*	*Non-smokers (N=1874)*	*OR (95% CI)*	*AOR (95% CI)*	*p*
Premature birth	11	107	2.93 (1.50–5.73)	2.976 (1.50–5.90)	0.002
Low birth weight	10	70	4.09 (2.01–8.31)	4.286 (2.07–8.88)	<0.001
Macrosomia	4	99	1.04 (0.37–2.91)	1.07 (0.38–3.01)	0.902
Growth restriction	6	99	2.66 (1.37–5.20)	2.702 (1.37–5.33)	0.004
Birth defect	5	47	2.86 (1.10–7.41)	2.664 (1.00–6.97)	0.049

AOR: adjusted odds ratio; adjusted for maternal age, education level, occupational status, first pregnancy status, and first birth status.

## DISCUSSION

Previous studies have shown that active and passive smoking during pregnancy significantly increase the risk of adverse pregnancy outcomes, such as preterm birth, low birth weight^12^, fetal complications^13^, and perinatal death^14^. This study confirms that active and passive smoking increase the risk of adverse pregnancy outcomes in Shanghai, China.

Our study revealed that the rate of passive smoking among pregnant women was 43.6%, which is higher than that in other countries^15^. Smoking is more common at home, at work and in public places, which may be related to social habits in China.

Active smokers had lower education level, with 84.9% having lower than a university degree, which is similar to findings from Jordan^16^, Iran^17^, and Slovakia^18^, suggesting that higher rates of smoking among pregnant women with lower education level are common worldwide. This could be for a variety of reasons. First, these women may have less access to information about the dangers of smoking in school and the workplace. Additionally, they may be more susceptible to social pressures and peer influence, making it easier for them to start smoking. Second, people with lower socioeconomic status are more likely to smoke. This may be because they face greater challenges in terms of employment, education, and other resources. Finally, some cultures may encourage smoking or have a lack of awareness about the dangers of smoking. In these communities, pregnant women with lower education level may be more susceptible to this social influence^6,19^.

Compared to non-smokers and passive smokers, active smokers had lower rates of first birth and first pregnancy status. This result is in accordance with most research results^16^. This could be primarily due to the awareness and cautiousness of these first-time mothers towards the potential harms of smoking, and these mothers are more likely to take measures to quit smoking.

We found that the newborns of active smokers had significantly earlier gestational ages and lower birth weights. This finding has been confirmed by several studies regarding the risk of premature birth and low birth weight in smokers^6,20^. To ensure the health of both mothers and babies, it is recommended that pregnant women avoid smoking during pregnancy and increase antenatal care and fetal monitoring to ensure the health and normal development of fetuses.

After adjusting for confounding factors, logistic regression analysis showed that passive smoking was a risk factor for preterm birth, low birth weight, and intrauterine growth restriction. Tobacco and smoke contain thousands of toxic and carcinogenic elements. When pregnant women are exposed to environments with tobacco and smoke, the harmful elements are absorbed through the respiratory system or skin, enter the blood circulation system, and cross the placental barrier, negatively affecting the growth and development of the fetus^21,22^. Therefore, to protect the health of mothers and children, pregnant women should avoid exposure to secondhand smoke.

Our study shows that active smoking during pregnancy was more harmful to maternal and infant health than passive smoking, which is consistent with previous research^12,16,23^. Studies have shown that newborns of pregnant women who actively smoke are more likely to be born preterm, have a low birth weight, and experience intrauterine growth restriction. Moreover, smoking also increases the risk of birth defects, such as heart malformations and neural tube defects, which negatively affect fetal health and development^24,25^. In the future, we need to further strengthen the creation of smoke-free environments. For example, the government should strengthen laws and regulations on tobacco control, increase public health awareness, and help more people to realize the harm of active and passive smoking. In addition, families, work units and communities should also actively promote the creation of smoke-free environments, encourage smokers to quit smoking, and provide healthy, smoke-free living environments for pregnant women.

### Strengths and limitations

The study had a large sample size of 3446 pregnant women, allowing a robust statistical analysis. The division of participants into active smokers, passive smokers, and non-smokers provides valuable insights into the differential effects of smoking on pregnancy outcomes. The comprehensive analysis of multiple pregnancy outcomes and the use of logistic regression to identify risk factors strengthen the conclusions.

The study has several limitations that should be acknowledged. Firstly, due to our exclusion criteria, we focused our analysis on a limited set of perinatal outcomes and did not consider pregnancy complications such as gestational diabetes, gestational hypertension, and others that may be associated with smoking. Secondly, the information regarding smoking behavior during pregnancy relied primarily on self-reporting, which is subject to potential biases such as underreporting or misreporting. Additional limitations include the small number of active smokers in our study population, which may have limited the statistical power to detect certain associations. Furthermore, the generalizability of our findings to other countries and populations may be limited due to differences in smoking patterns, healthcare systems, and other factors.

## CONCLUSIONS

We investigated the active and passive smoking status of pregnant women and the effects of smoking on pregnancy outcomes. The results show that active and passive smoking increased the risk of adverse pregnancy outcomes. This study provides data on the relationship between maternal smoking during pregnancy and birth outcomes, and can help professionals develop more effective strategies to address these issues, thereby improving the health of pregnant women and their newborns.

## Data Availability

The data supporting this research cannot be made available for privacy or other reasons.
